# A stepped wedge, cluster controlled trial of an intervention to improve safety and quality on medical wards: the HEADS-UP study protocol

**DOI:** 10.1136/bmjopen-2014-007510

**Published:** 2015-06-22

**Authors:** Samuel Pannick, Iain Beveridge, Hutan Ashrafian, Susannah J Long, Thanos Athanasiou, Nick Sevdalis

**Affiliations:** 1 NIHR Imperial Patient Safety Translational Research Centre, Imperial College London, London, UK; 2 West Middlesex University Hospital NHS Trust, London, UK; 3 Department of Surgery & Cancer, Imperial College London, London, UK; 4 St Mary's Hospital, Imperial College Healthcare NHS Trust, London, UK; 5 Centre for Implementation Science, Health Service & Population Research Department, King's College London, London, UK

**Keywords:** GENERAL MEDICINE (see Internal Medicine)

## Abstract

**Introduction:**

The majority of preventable deaths in healthcare are due to errors on general wards. Staff perceptions of safety correlate with patient survival, but effectively translating ward teams’ concerns into tangibly improved care remains problematic. The Hospital Event Analysis Describing Significant Unanticipated Problems (HEADS-UP) trial evaluates a structured, multidisciplinary team briefing, capturing safety threats and adverse events, with rapid feedback to clinicians and service managers. This is the first study to rigorously assess a simpler intervention for general medical units, alongside an implementation model applicable to routine clinical practice.

**Methods/analysis:**

7 wards from 2 hospitals will progressively incorporate the intervention into daily practice over 14 months. Wards will adopt HEADS-UP in a pragmatic sequence, guided by local clinical enthusiasm. Initial implementation will be facilitated by a research lead, but rapidly delegated to clinical teams. The primary outcome is excess length of stay (a surplus stay of 24 h or more, compared to peer institutions’ Healthcare Resource Groups-predicted length of stay). Secondary outcomes are 30-day readmission or excess length of stay; in-hospital death or death/readmission within 30 days; healthcare-acquired infections; processes of escalation of care; use of traditional incident-reporting systems; and patient safety and teamwork climates. HEADS-UP will be analysed as a stepped wedge cluster controlled trial. With 7840 patients, using best and worst case predictions, the study would achieve between 75% and 100% power to detect a 2–14% absolute risk reduction in excess length of stay (two-sided p<0.05). Regression analysis will use generalised linear mixed models or generalised estimating equations, and a time-to-event regression model. A qualitative analysis will evaluate facilitators and barriers to HEADS-UP implementation and impact.

**Ethics and dissemination:**

Participating institutions’ Research and Governance departments approved the study. Results will be published in peer-reviewed journals and at conference presentations.

**Trial registration number:**

ISRCTN34806867.

**Strengths and limitations of this study**

- General wards typically generate the errors that lead to preventable deaths, but we know relatively little about how to improve the safety of care in this specific setting.
- This study will evaluate a new strategy to incorporate proactive team risk surveillance into routine care on general medical wards, with a facilitated organisational response: Hospital Event Analysis Describing Significant Unanticipated Problems (HEADS-UP).
- Mixed methods (quantitative and qualitative) will identify different aspects of the impact of HEADS-UP.
- With a relatively prolonged data collection period, the study is prey to unanticipated changes affecting participating sites.

## Background

### Patient safety on medical wards

Despite an intense focus on healthcare safety in recent years, medical wards remain potentially perilous. Over 60% of medical wards’ failings reach their patients, and 10% of those failings cause physical injury.^1^ These are not trivial problems: the general ward, more than any other care setting, generates the errors that lead to preventable deaths.^2^ Technical procedural failings are infrequent; more often, teams struggle to reliably monitor, assess and reassess their patients.^2^ Medical teams face increasing workloads,^3^ and concerns about the basic processes of ward care have been well publicised.^4^

Nonetheless, there is a relative paucity of specific literature on how to improve the quality and safety of care on medical wards. The same factors that contribute to serious errors in this setting—heterogeneous patient populations, geographic dispersion of medical teams, and frequent changes in staff, policies and procedures—have hampered the research necessary to address them. As a result, quality and safety interventions for medical patients are largely extrapolated from more structured clinical environments, that is, operating theatres and intensive care units (ICUs). Staff in medical units, however, would rapidly attest to the different challenges they face, compared to their colleagues in these other areas.

A recent review by our group established that interventions to improve medical ward care cluster across five themes:^5^

- Improving staffing levels and team composition;
- Improving communication and collaboration;
- Standardising care processes;
- Early recognition and treatment of the deteriorating patient;
- Improving patient safety climate.

Although the evidence base underpinning them is fragmented, these interventions are likely to have some positive impact on the quality and safety of care. Organisations increasingly adopt multifaceted improvement strategies that incorporate combinations of these five themes.^6^ ^7^ These complex strategies are highly specific to their setting, and may be costly to implement.^8^ This limits their wider applicability, and a search for a more universally relevant tool is warranted.

### Frontline ward staff: a knowledgeable, but underused, source of information

Frontline staff are a relatively untapped source of information about their own organisation and the effectiveness of its procedures and processes. It matters, what staff say: their perceptions correlate with patient survival.^9^ Moreover, finding the right tools to elicit staff concerns might facilitate change: organisations that seek out discomfiting insights, consciously listening to their staff, develop more holistic improvement strategies.^10^ Still, there are few descriptions of successful tools that systematically capture staff knowledge to improve clinical processes. Centralised incident reporting systems have major failings,^11^ ^12^ and physicians as a professional group do not engage with them.^11^ Improving reporting systems is a laudable goal, but additional methods are required to adequately detect and address problems before they lead to adverse events and patient harm.^13^

Importantly, the problem may lie with the available tools themselves, not their users. Ward staff are willing to disclose concerns about errors and potential errors to their peers,^14^ and incident reporting rates increase when clinicians are given more reporting options and receive feedback.^15^ Interactive schemes, visibly engaging entire teams to identify recurrent problems, may complement more traditional systems relying on individual reports.^16^ ^17^ There is increasing interest in novel strategies to take advantage of team knowledge: for medical teams, the ward round may represent a specific opportunity to identify patient safety issues.^18^ Moreover, studies in paediatric centres suggest that patient-relevant process measures improve with a structured team approach to risk monitoring and situational awareness (eg, ‘team huddles’).^19^ ^20^ It seems that a collective awareness of safety problems exists; exploiting it may have real benefits, beyond the typical reliance on individual efforts to intercept a catastrophic clinical deterioration, or report it after the event.

In our review,^5^ we identified no reports of simple interventions which (1) specifically targeted adult medical wards; (2) leading to team-wide engagement in a quality and safety initiative; with (3) rigorous assessment of patients’ clinical outcomes. More complex interventions in this field have been tailored to the specific needs of individual clinical trial sites, or require resource-intensive support and mentoring,^21^ which limit their uptake in wider practice. Moreover, there are few reports of community hospitals replicating the experience of academic centres.^22^

The HEADS-UP (Hospital Event Analysis Describing Significant Unanticipated Problems) study that we describe here addresses these limitations with a tool that can be rapidly and easily adopted in any healthcare context. Our intervention aims to identify and mitigate systematic failings through daily whole-team recognition of clinical risks, facilitating improved team situational awareness, allied to a rapid organisational response.

### The HEADS-UP intervention

**Figure 1:**
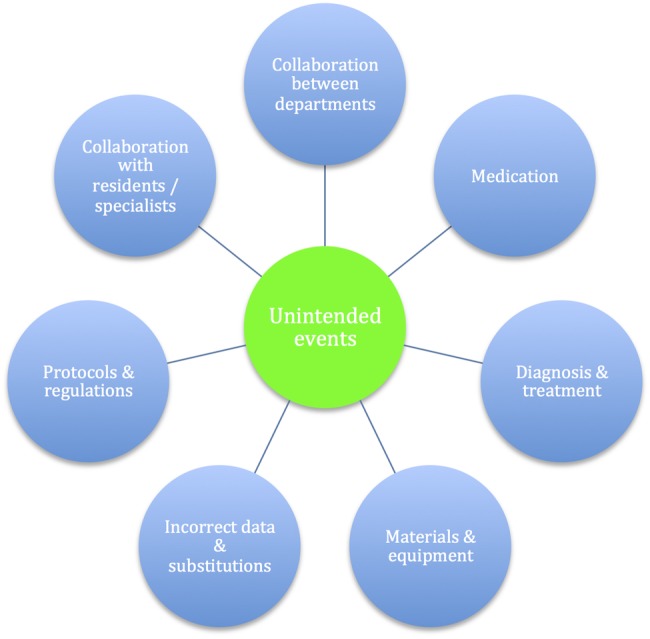
Categories of unintended events in internal medicine departments impacting negatively on patient care (after Lubberding *et al*^1^).

**Figure 2:**
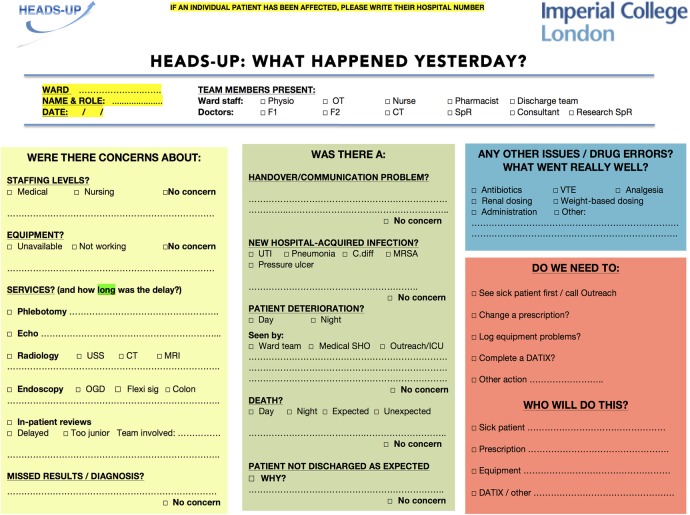
The HEADS-UP (Hospital Event Analysis Describing Significant Unanticipated Problems) team briefing tool.

A prompt-led team briefing (HEADS-UP) was designed to help multidisciplinary medical ward teams discuss clinical and administrative challenges, including adverse events, of the preceding 24 h. The briefing can be led by any member of the team, regardless of seniority or role. HEADS-UP identifies ongoing concerns amenable to immediate intervention, as well as those requiring more detailed assessment and input from other departments. It also prompts team members to share information that they may not volunteer spontaneously.^23^ ^24^ Categories of prompts were guided by the published literature on the nature of common unintended events in medical departments^1^ (figure 1), with additional suggestions from ward clinicians regarding frequent or potentially serious lapses in care noticed in their clinical areas. At the request of participating physicians, a checklist-style pro-forma was used to facilitate speed of completion. We adopted a visual format similar to the World Health Organization's surgical safety checklist (figure 2). HEADS-UP summaries are regularly disseminated to participating teams, senior clinicians, managers and executives.

### Study aim and hypotheses

The primary aim of this study is to assess the impact of the HEADS-UP briefing tool on clinically relevant patient outcomes. The secondary aim is to explore how changes in patient outcomes, if any, are mediated by changes in workplace climate and ward processes.

We hypothesised that:

- Team use of the HEADS-UP briefing would empower junior clinicians to voice concerns, improving their teams’ situational awareness (an important factor in mitigating risks^19^) and their units’ safety and teamwork climates;
- This would promote early team recognition of the deteriorating patient, and facilitate the process of escalation of care;
- Information generated by ward teams would both inform their own practice and prompt downstream service reorganisation;
- The combination of ward and support service improvement would improve clinical outcomes, with a dose-response relationship (ie, the better the tool is used in practice, the greater the benefit seen);
- An explicit focus on team-wide recognition of adverse events would improve engagement with existing incident-reporting systems, thus leading to an increase in formally reported incidents within wards implementing HEADS-UP.

## Methods and design

### Study design and setting

**Figure 3:**
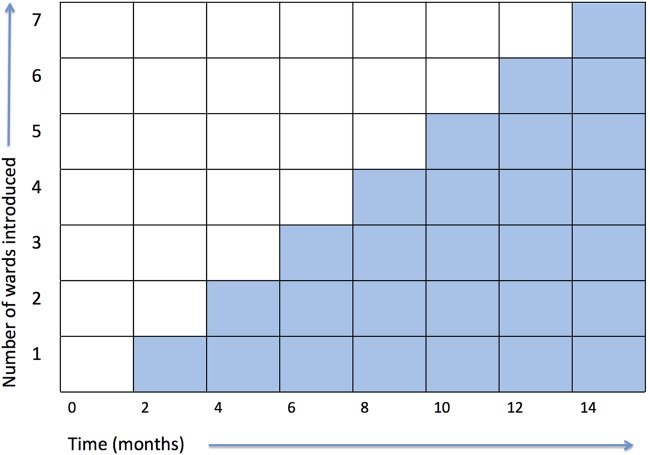
HEADS-UP (Hospital Event Analysis Describing Significant Unanticipated Problems) stepped wedge cluster design.

HEADS-UP is a prospective stepped wedge, cluster controlled trial, conducted in two hospitals in London, UK. The stepped wedge design involves the sequential introduction of the intervention to each of the clusters (in this case, wards) over time, with clusters progressively moving from the control group to the intervention group (figure 3). The staged implementation facilitated by the stepped wedge design is particularly helpful when simultaneous rollout of an intervention to all clusters is impractical, for example, for logistical or financial reasons. This trial design is used to evaluate interventions whose effects are predicted to be more beneficial than harmful, especially those interventions embedded in daily clinical practice.^25^ Stepped wedge designs are increasingly used in trials of interventions in acute care.^26–29^

The order in which clusters receive the intervention will be guided by logistical restrictions, and the de facto recognition of clinicians enthusiastic to introduce HEADS-UP to their wards. Although cluster randomisation (randomising the order in which the clusters receive the intervention) would be preferable, it is important to recognise the impact of willing early adopters who then lead their colleagues in implementing the intervention.^30^ They are likely to participate more extensively and follow through more rigorously and enthusiastically with the intervention than units at later stages of the intervention diffusion.^31^ Sufficient leadership and support from these early adopters will be needed to maximise the use of HEADS-UP in all the desired areas, much as the introduction of surgical checklists, for instance, has historically relied on strong clinical leadership.^32^ HEADS-UP is introduced to new clusters at two-monthly intervals.

The study is conducted at two sites. The first is a university-associated community general hospital. Clusters from this site are generated from the acute admissions and downstream medical (gastroenterology, respiratory and geriatric) wards. Each cluster comprises clinical areas that are physically linked, served by the same medical team, or both. This will help to limit contamination between groups. The second site is an academic hospital, where HEADS-UP will be implemented within the geriatric wards.

### Study population

The study focuses on adult medical patients admitted to study wards between 2013 and 2015. To isolate the effect of the intervention, patient-level exclusion criteria will include:

- Time spent on the specified ward comprising less than 50% of the total inpatient stay;
- Discharge to a new skilled care facility or other hospital (ie, not the patient's address at the time of admission; discharge to a new facility typically incurs substantial delays, outside of the ward team's control);
- Multiple intrahospital ward transfers. A single transfer from the initial admissions unit to a downstream medical ward is permitted. One further transfer to an escalation area to facilitate discharge (whereby the patient spends less than 24 h in the escalation area immediately prior to their discharge home) is also permitted;
- Admission to the high-dependency unit, or ICU;
- Elective admission or direct admission from another hospital;
- Surgeon-directed care for more than 24 h during the inpatient stay.

### Outcome measures

*Primary outcome*

- Excess length of stay (eLOS)—a surplus stay of 24 h or more, compared to peer institutions’ Healthcare Resource Groups-predicted length of stay.

*Secondary outcomes*

- eLOS or 30-day readmission;
- In-hospital death or death/readmission within 30 days;
- Complications of care: hospital-acquired infections and pressure ulcers;
- Processes of escalation of care: use of the ICU outreach service, admissions to the ICU, and cardiac arrest calls;
- Staff engagement with the traditional web-based reporting system: number, and type (eg, patient falls or communication failures) of formally reported incidents;
- Patient safety climate: teamwork and safety subsections of the well validated Safety Attitudes Questionnaire (SAQ).^33^

We believed that tangible improvements in clinical outcomes and quality of care would be mediated by changes in ward processes, and ward safety and teamwork climates. Our chosen outcomes allow us to evaluate this hypothesis. The selected measures are similar to those used in other studies evaluating the quality of ward care,^34^ and have been assessed in a UK setting where appropriate.^33^ Table 1 describes specifically how the components of these outcomes correspond to the study hypotheses outlined earlier.

**Table 1:** Study outcomes and corresponding hypotheses evaluated within the HEADS-UP trial

	Outcome component	Relevant hypothesis	Rationale for outcome selection
Primary outcome	Excess length of stay	Improved clinical outcomes through ward and support service improvements	Length of stay reflects efficient resource use, and possibly quality of care.^35^ Length of stay varies substantially within institutions, with wide differences between the acute admissions unit and downstream wards. Using excess length of stay as an outcome increases study power, facilitating statistical detection of a meaningful change in outcome without requiring an excessive number of wards or data collection period.

Secondary outcomes: clinical outcomes	Mortality	Improved clinical outcomes through ward and support service improvements	Correlates with quality of care^36^ and may relate to performance in non-technical skill domains.^37^
	Readmission	Improved clinical outcomes through ward and support service improvements	Need to confirm that improvements in hospital efficiency do not come at the expense of increased readmissions. 37% of medical readmissions are avoidable,^38^ a proportion that can be reduced with targeted quality improvement initiatives.^39–41^
	Complications of care	Improved situational awareness will mitigate patient risks	Agreement that these outcomes are appropriate patient safety indicators.^42^ Reliable reporting of these outcomes to confirm adherence to stringent centrally mandated targets.^43^ ^44^

Secondary outcomes: processes of care	Escalation of care	Earlier team recognition of the deteriorating patient will facilitate processes underpinning escalation of care	Multidisciplinary interventions, increasing team situational awareness,^45^ may address staff reluctance to appropriately escalate their concerns.^24^ ^46^

Secondary outcomes: staff outcomes	Staff engagement with traditional reporting system	Team-wide recognition of adverse events will improve engagement with existing incident reporting systems	More reports overall, with a lower contribution from reports of slips and falls, are associated with more positive safety culture and risk management ratings.^47^
	Safety and teamwork climate	Empowerment of junior clinicians, with structured communication tool, will improve perceptions of safety and teamwork	Improved safety climate is associated with organisation-wide reduction in adverse events.^48^ SAQ scores previously assessed both in inpatient settings and in the UK.^33^

HEADS-UP, Hospital Event Analysis Describing Significant Unanticipated Problems; SAQ, Safety Attitudes Questionnaire.

### Intervention implementation

#### HEADS-UP implementation at ward level and clinical engagement

Multidisciplinary ward teams will be asked to use HEADS-UP on a daily basis during the normal working week (Monday to Friday). Staffing and service provision are significantly reduced out of hours, and it was not deemed practical to incorporate a HEADS-UP briefing at night or at weekends within those constraints. One of the study leads at each site will supervise the initial use of the tool and answers any questions arising from it. The presence of a researcher will be documented when appropriate.

To maximise clinical engagement with this intervention, we aim to disrupt teams’ existing working patterns as little as possible; they will use HEADS-UP wherever it fits most naturally into their existing schedule. We anticipate that this will ordinarily be early in the day. The HEADS-UP tool itself acts as the written record of the briefing. Its format will remain stable, but further minor changes will be made at the request of individual ward teams to make it responsive to their specific needs. Successful improvement interventions reported elsewhere have adopted a similarly flexible approach.^32^ ^49^

Support for the study will be sought from senior clinicians and executives with responsibility for clinical quality, safety and risk management at each site. Presentations to individual clinical teams will publicise the intervention prior to its introduction. No protected time will be available for HEADS-UP training, but the ideal format for each briefing will be discussed in departmental rounds, team meetings and with participating clinicians. The information shared during the daily HEADS-UP briefings may prompt a degree of reflective practice and immediate learning. In addition, a regular summary of the HEADS-UP events recorded from their clinical area will be given to each team. The format of this feedback again depends on the team's existing schedule; where possible, it will be incorporated into existing departmental educational or governance meetings, to place it in the appropriate context and minimise any additional time commitments. This feedback will emphasise the ongoing impact of the information gathered during the HEADS-UP briefings, highlighting any subsequent quality improvement work or changes to support services.

HEADS-UP summaries will also be shared with the governance committees responsible for the issues raised. Clinicians and managers already accountable for quality and safety in these clinical areas will be expected to use the information appropriately to guide resource allocation, and make changes to routine processes or procedures as they see fit. No specific guidance will be issued as to how the HEADS-UP data will be used. We aim to evaluate how, in practice, the intervention will generate changes in clinical services. However, specific safety concerns and significant adverse events raised through HEADS-UP briefings will be emphasised to the responsible clinical team or governance body in order that they take appropriate action.

#### Fidelity of implementation and confounding factors

The quality of HEADS-UP implementation will be evaluated primarily through the daily briefing documentation, recording the team members present, number and type of concerns raised, and the decisions taken as a result. A narrative diary, describing the qualitative impact of HEADS-UP, as well as the downstream quality improvement work and service changes arising from it, will complement the quantitative outcomes. The narrative account will also describe the observation of a number of HEADS-UP briefings, and the extent to which they held true to the perceived ideal in terms of participants, timeliness, focus and intent. The individual, team and organisational barriers and facilitators to the effective implementation of HEADS-UP will be assessed. Together with the objective outcomes listed in table 1, and the staff survey (SAQ) data, this will complete a mixed methods analysis of the HEADS-UP programme.

Staff workload and patient casemix are likely to be the two predominant confounding factors for this non-randomised study. Ward bed occupancy rates will be documented from routine hospital administration systems, and staff perceptions of workload assessed periodically with the validated NASA-Task Load Index (NASA-TLX) tool.^50^ Patient casemix will be tracked with an updated version of the Charlson comorbidity index.^51^ ^52^

#### Blinding

Professionals implementing the intervention are not blinded to the ward's assignment group: this is not possible, given the nature of the intervention. Extraction of the clinical outcome measures will be performed primarily by administrative staff not involved in the study (as part of their ordinary duties), who will be blinded to intervention groups.

### Data management

Data will be extracted directly from hospital administrative systems, with a monthly assessment by administrative staff to confirm its reliability. Anonymised data, where appropriate, will be held securely on password-protected hospital intranet systems. Given the nature of the intervention, the time scale of the study, and the extraction of outcomes from existing administrative systems, no data monitoring committee is required. The summarised trial data set will be held by SP, and disseminated to local clinical and managerial healthcare providers as required after the study, with no contractual limitations.

### Sample size, power calculation and analysis

In the 33 months preceding the study, local data from six of the seven anticipated clusters described a median of 522 patients matching our inclusion/exclusion criteria each month (range 316–722). Mean eLOS rates ranged from 5.6% to 51.5%.

Cluster controlled trials also require an estimation of the intraclass correlation coefficient (ICC); this is complex, and observed ICCs rarely match their predicted values. ICC estimation should take into account the results of previous studies, as well as the anticipated numbers of individuals and clusters in the index study. Greater numbers of individuals in a study reduce the width of the ICC confidence interval, mitigating the effect of a relatively small number of clusters.^53^ In addition, clinical outcome measures tend to have lower ICCs than process measures.^53^ The observed ICC for falls in a multifactorial intervention on elderly care wards was only 0.007.^54^ However, ICCs for length of stay and appropriateness of stay in trials of inpatient care pathways were an order of magnitude higher.^55^ We therefore adopted the more conservative ICC estimate of 0.06. In any case, the power under a stepped wedge cluster randomised trial is relatively insensitive to ICC underestimation, compared to a parallel cluster design.^56^ Similarly, stepped wedge trial power is relatively insensitive to variations in the coefficient of variation.^57^ In addition, in our best-case prediction the coefficient of variation approached the level at which adjusting for variable cluster size has a negligible impact on sample size, even in parallel cluster trials.^58^ No adjustment for coefficient of variation was therefore required.

Given the variation in baseline outcomes in our local data set, we then estimated the study's power to detect a 1 standard deviation reduction in eLOS on the wards with the highest and lowest baseline outcome rates. With 7840 patients in the trial (560 patients/month), and two-sided p<0.05, the study would achieve 100% power to detect a 14% absolute risk reduction. At worst, in the ward with the lowest baseline outcome rate, it would achieve 75% power to detect a 2.3% absolute risk reduction. We therefore propose that the study's power to detect a 2–14% absolute risk reduction lies between 75% and 100%. With this complex trial design, statistical uncertainty in power calculations is not unusual. The recent protocol of a large stepped wedge cluster randomised trial similarly produced a range within which its power might lie.^29^ Experts also advocate more widespread recognition of the inherent limitations of statistical power thresholds.^59^

The stepped wedge design compares outcomes in each cluster before and after the introduction of the intervention. Overall differences in outcomes between preintervention and postintervention periods will be reported. Primary analysis will be on an intention-to-treat basis, with a separate prespecified per-protocol analysis of those units implementing the intervention with high fidelity. Analyses will use the patient-level data described above, clustered within ‘units’, using random effects to model the correlation between individuals within the same cluster. Generalised linear mixed models, or generalised estimating equations, will form the basis of the analysis.^57^ Results will also be assessed using a time-to-event regression model. Underlying temporal trends (including seasonal trends) will be accounted for. We will evaluate any interaction effect between intervention group and ‘duration of HEADS-UP implementation’ to see if the intervention exerts an incremental effect over time. There will be a prespecified analysis excluding patients coded for palliative care, to further isolate the effect of the intervention. Sensitivity analyses will judge whether primary outcome results remain unchanged when other patient groups (eg, those spending less than 50% of their stay on the specified ward) are included. No interim analyses are planned, nor is a formal health economics analysis.

## Results: pilot implementation

During a 6-week pilot period, participating clinicians honed the format of the HEADS-UP tool, ensuring it could be completed quickly and accurately. Subsequent iterations of the HEADS-UP tool incorporated further feedback from clinical staff and clinical governance teams on the content and use of the HEADS-UP briefing. This largely resulted in an increased focus on the outcomes of the briefing, namely the actions to be taken and which team member would be responsible for them.

The introduction of the tool was supervised initially by a physician researcher (SP). When led by a clinician, the HEADS-UP briefing typically took between 5 and 8 min to complete. Clinical teams’ use of the tool was deliberately unsupervised for the latter half of the pilot period, to gauge whether it was suitably concise and relevant to be used in practice without the presence of the researcher. The HEADS-UP briefing was completed unsupervised on 80% of working days. Taken together, these data suggest that the HEADS-UP trial is feasible.

## Trial status

Data collection is ongoing.

## Discussion

This stepped wedge cluster controlled trial will assess the impact of a simple intervention to improve quality and safety on medical wards. The design of the HEADS-UP tool, as well as its proposed implementation, intentionally minimises the disparity between the trial setting and daily clinical practice. This will make the study results immediately applicable to broad swathes of healthcare settings. We hope to obtain conclusive evidence for the success or failure of the intervention, but with a relatively prolonged data collection period, the study is prey to unanticipated systemic changes outside of our control. There is also a tension between effective HEADS-UP promotion, essential for adequate staff engagement, and contamination of the control groups. In addition, downstream interventions arising from the HEADS-UP process may impact multiple wards, regardless of their participation in HEADS-UP. The narrative record will help highlight where this may have been the case, and contextualise the impact of the tool.

In summary, HEADS-UP offers a novel, rapid whole-team analysis of clinical and administrative challenges, including adverse events, at ward level. This prospective trial will identify whether HEADS-UP is a useful addition to existing safety systems, and broader lessons for the implementation of safety and quality interventions within the complex medical ward environment.
